# Size-Exclusion Chromatography of Macromolecules: A Brief Tutorial Overview on Fundamentals with Computational Tools for Data Analysis and Determination of Structural Information

**DOI:** 10.3390/polym17050582

**Published:** 2025-02-22

**Authors:** José Ginés Hernández-Cifre, Mar Collado-González, Francisco Guillermo Díaz Baños, José García de la Torre

**Affiliations:** 1 Department of Physical Chemistry, University of Murcia, 30100 Murcia, Spain; fgb@um.es (F.G.D.B.); jgt@um.es (J.G.d.l.T.); 2 Department of Cellular Biology and Histology, University of Murcia, 30100 Murcia, Spain; mdmcg1@um.es

**Keywords:** chromatography, computation, molecular weight, size exclusion, structure

## Abstract

Size-exclusion chromatography (SEC) is presently a widely used and very informative technique for the characterization of macromolecules in solution. Beyond the first implementations of SEC—which required cumbersome column calibrations and were mainly intended for the determination of molecular weights—the modern SEC approach involving multiple detectors (md-SEC) is based on solution properties such as intrinsic viscosity and light scattering. Thus, md-SEC enables the direct and more efficient determination of molecular weights, as well as the determination of relationships between property and molecular weight, which can be quite useful in structural studies. Here, we first present a review of the fundamental aspects of the dilute-solution properties of macromolecules—particularly the differential refractive index, intrinsic viscosity, and scattering-related properties—on which the various detectors involved in md-SEC are based. Then, we developed SECtools, a suite of public-domain, open-source computer programs, which allow for the full analysis of md-SEC chromatograms. These analyses range from just the recorded raw signals (mV) of the detectors to a full determination of molecular weight averages and distributions. The use of these programs is illustrated through experimental studies using various samples.

## 1. Introduction

The most essential aspect of the characterization of macromolecular compounds is the determination of their molecular weight, *M*. Furthermore, in many cases, the sample is polydisperse, containing macromolecules with various (a few or many) species with varying *M*. Then, the characterization would consist of the determination of the molecular weight distribution or, at least, the molecular weight averages and degree of polydispersity. Another essential aspect is the elucidation of the global conformation (shape or flexibility) of the macromolecular chains, which is usually performed by studying the molecular weight dependence of properties in solution—either hydrodynamic quantities such as the intrinsic viscosity, [η], or other quantities obtained from scattering techniques like the radius of gyration $R_{g}$ [1,2].

These purposes require the fractionation of the sample into some number of fractions with different (narrower) ranges of size, which would be later individually characterized to obtain some estimate of their approximate or average *M*, typically from static scattering or from some known property vs. *M* relationship, such as the [η] vs. *M* Mark–Houwink–Sakurada (MHS) equation. In the earliest days of polymer science, fractionation was laboriously carried out by fractional precipitation, based on the *M*-dependence of the solubility of polymers in poor solvents. In subsequent steps, portions of a non-solvent were added to the polymer solution in order to cause the precipitation of fractions of polymers with successively decreasing *M* values. Later, advances in chromatographic techniques made it possible to develop size-exclusion chromatography (SEC) fractionation by passing the polymer solution through a column containing a material—such as a gel—through which the polymer molecules permeate at a given rate, depending on their *M*. The fractions thus separated by gel-permeation chromatography (GPC) were collected and individually characterized.

More recently, this preparative SEC/GPC chromatography approach has been completed with online detectors. First, a concentration-sensitive detector was added to measure the concentration of the eluting solute as a function of the elution volume. This allowed for the determination of the molecular weight distribution with a previous ad hoc calibration of the column [3]. Then, the miniaturization of light scattering and viscosimetric instrumentation provided the online measurement of *M* and [η]; furthermore, other detectors are now available to measure ultraviolet–visible absorbance, dynamic light scattering (DLS), and even small-angle X-ray scattering (SAXS).

The importance of multi-detector size-exclusion chromatography (hereafter, md-SEC) has been recognized in textbooks [1,2,4] and monographs [3,5,6]. While primitive md-SEC instrumentation was somehow modular, even perhaps in-house-assembled, most modern instruments are compact and governed by vendor-provided, sophisticated software. These setups allow for efficient data acquisition and analysis, which are indeed most convenient for routine work. The present work is motivated by the interest that is present in academic and research laboratories in having software tools with open sources, particularly for the task of data analysis. The understanding of the principles of md-SEC would provide confidence regarding the use and application of this technique. Indeed, such principles serve as the basic core of the physical chemistry of macromolecules in solution, and knowledge about them and how they are implemented in computer programs would be of pedagogic value. These opinions motivated the present article, in which we describe fundamental aspects and their implementation in the source codes of public domain programs, and provided some guidance for the interpretation of the results.

## 2. Fundamentals

In this section, we give a brief description of SEC separation and the primitive modes of data analysis based on column calibration. Then, we present the basic theory of macromolecular solution properties on which the detector modes—particularly light scattering (LS) and intrinsic viscosity (VIS)—are based.

### 2.1. SEC Separation and Primary Calibration Modes

**Figure 1:**
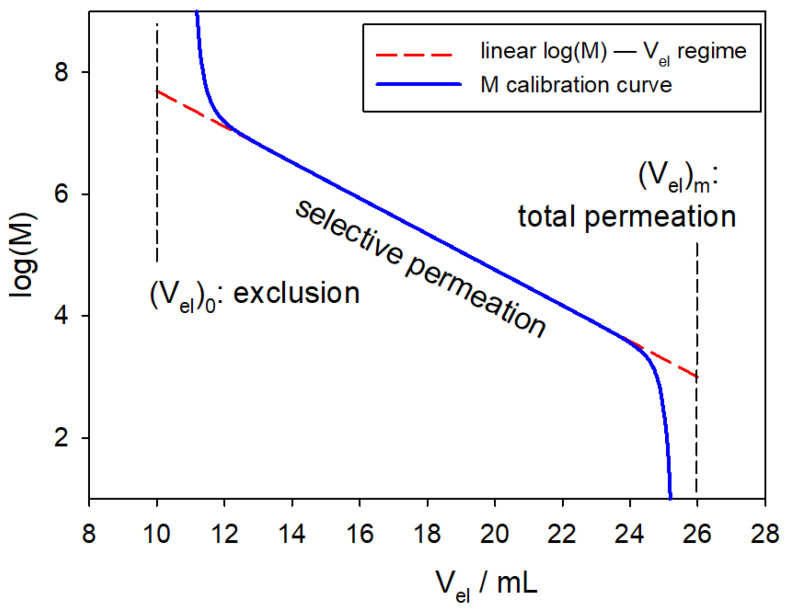
Typical logM vs. $V_{el}$ calibration curve (see, e.g., [7]), with ${\left(V_{el}\right)}_{0}\approx 10$ mL, ${\left(V_{el}\right)}_{m}\approx 26$ mL, $M_{high}=5\times 10^{7}$ Da, and $M_{low}=1.0\times 10^{3}$ Da. Molecules with $M>5\times 10^{7}$ Da are not retained at all and are eluted altogether at $(V_{el})\approx 10$ mL. Molecules with $M<1.0\times 10^{3}$ Da are all fully retained and are eluted altogether at ${\left(V_{el}\right)}_{m}\approx 26$ mL. In the regime of selective permeation, $V_{el}$ is linearly related to logM.

The basic principle in SEC is the relationship between the molecular size of the species composing the solute and the retention volume (i.e., the elution volume at which they emerge, $V_{el}$). Under some circumstances, particularly when the solute is a polydisperse polymer with molecules that differ only in molecular weight, the size is dictated only by the molecular weight of the species, *M*. The relationship between *M* and $V_{el}$ usually follows the trend displayed in Figure 1. The largest molecules, with *M* being above some approximate threshold value, $M_{high}$, are not retained at all and are eluted altogether at ${\left(V_{el}\right)}_{0}$. Conversely, the smallest molecules, with *M* being below another approximate threshold value, $M_{low}$, are all fully retained and their elution requires a limiting value such that they are eluted altogether at ${\left(V_{el}\right)}_{m}$. In the regime of selective permeation, $V_{el}$ is linearly related to logM.

A basic SEC instrument is a single-detection chromatograph with only one mass detector whose signal, at any time after the passage of a volume $V_{el}$ of the eluent, would detect the presence of the solute. The instrument should be, first of all, calibrated to determine a $V_{el}$–*M* curve, like that in Figure 1. For instance, in the study of a sample of poly(styrene), PS, one would first perform runs for samples of PS of known molecular weight, thus determining a series of ($V_{el}$, *M*) data points to construct the calibration curve. Then, for the problem sample, in a chromatogram measured in the same conditions, one would measure $V_{el}$ to determine the corresponding *M* from the calibration curve.

A landmark in the SEC field was the proposal of the so-called universal calibration by Benoît et al. [7,8]. According to it, a unique curve of log(M[η]) vs. $V_{el}$ may serve for all the macromolecular compounds within a common chemical family. For instance, a unique logM vs. $V_{el}$ calibration curve can be built for most vinyl polymers, even with different chain topologies (linear, star, brush, or randomly branched). The concept behind this important proposal—which is overlooked or incompletely explained in some sources—comes from Einstein’s viscosity theory of polymer solutions, which is intimately related to the volume of the solute particles. The Einstein fundamental equation states that the solution viscosity, η, in dilute solution, which is higher than that of the solvent, $\eta_{0}$, is given by $\eta =\eta_{0}\left(1+\nu \phi \right)$, where ν is a numerical constant (ν=5/2 for a spherical particle) and ϕ is the volume fraction of the solute particles [9]. In terms of the mass concentration *c* and an equivalent hydrodynamic volume of the particles $V_{h}$ (e.g., the hydrated volume of a globular protein, or the volume of the so-called Flory’s equivalent sphere [10,11,12] for a random coil polymer, which encompasses its surrounding, non-draining solvent molecules), Einstein’s equation reads $\eta =\eta_{0}\left(1+\nu cV_{h}/M\right)$. In terms of the intrinsic viscosity (see next section), this leads to $V_{h}=\left(1/\nu \right)M\left[\eta \right]$. Then, in the SEC/GPC column, the behavior (shorter or longer retention) depends on how $V_{h}$ compares to the range of volumes of the pore size of the gel in the column.

The advent of modern detectors in md-SEC made it possible to obtain properties such as the molecular weight and the intrinsic viscosity, directly, without calibration of the column, for any solute, as well as the amount of the analyte—the macromolecular solute—for the consecutive slices (i.e., each elution volume $V_{el}$) in the chromatogram.

### 2.2. Solution Properties

The successive fractions of the sample that are eluted from the column pass through a detection system that monitor some signals from them. The chromatogram consists of a set of (signal) _j_ values of the detectors vs. the elution volume ${\left(V_{el}\right)}_{j}$, corresponding to the *j*-th slice or fraction eluted between ${\left(V_{el}\right)}_{j}$ and ${\left(V_{el}\right)}_{j+1}$. The signals depend on properties associated with the solute of the fraction that is eluting. Some basic properties are those related to the amount of solute (i.e., to its concentration) in such slices, $c_{j}$ (for simplicity in the notation, we omit momentarily the *j* subscript). Such is the case for the refractive index increment, dn/dc. For a dilute solution, the increase, Δn, in the refractive index of the solution, *n*, with respect to that of the solvent, $n_{1}$, is given by

(1) $$\Delta n=n-n_{1}=\frac{dn}{dc}c$$

Another property—which, similarly to Δn, depends only on the concentration and chemical nature of the solute in each slice—is the absorbance at a fixed wavelength, related to the concentration through the extinction coefficient.

In md-SEC, the most important detectors are those that monitor properties which also depend on other features of the molecules, namely their mass, size, and structure. This is the case for light scattering (LS). According to conventional LS theory, as described in textbooks (see, e.g., [1,2,4,13,14]), the excess intensity scattered by the solute is given by the increase, $\Delta I=I-I_{solvent}$, in the light scattered by the solution, *I*, with respect to that for the pure solvent, $I_{solvent}$. According to the basic theory,

(2) $$\frac{Kc}{\Delta R}=\frac{1}{M}\frac{1}{P(\theta )}+2A_{2}c$$

In this equation, scattering intensities appear in the form of Rayleigh ratio, $R=Ir^{2}/I_{0}$, where $I_{0}$ is the intensity of the incident light and *r* is the distance from sample to detector. Therefore,

(3) $$\Delta R=(r^{2}/I_{0})\Delta I$$

In Equation (2), *K* is the so-called optical constant, defined as

(4) $$K=\frac{4\pi^{2}}{N_{A}\lambda_{0}^{4}}n_{1}^{2}{\left(\frac{dn}{dc}\right)}^{2}$$

where $\lambda_{0}$ is the wavelength of light in vacuum. The so-called form factor of the solute molecules, P(θ), can be approximately given by [1,2]

(5) $$\frac{1}{P(\theta )}\approx 1+\frac{q^{2}R_{g}^{2}}{3}$$

where the angular variable $q=\left(4\pi n/\lambda_{0}\right)\sin\left(\theta /2\right)$ is determined by the scattering angle θ, and $R_{g}$ is the radius of gyration, which depends on the size and conformation of the solute molecules. As a rule of thumb, it can be accepted that Equation (5) is valid when the second term in the right-hand side is sufficiently smaller than unity—e.g., $q^{2}R_{g}^{2}/3<0.5$. Furthermore, in Equation (2), the second term in the right-hand side, proportional to concentration, contains the second virial coefficient, $A_{2}$. In SEC, the solute, which is already diluted in the injected sample (typically 0.1 mg/mL), is further diluted as it appears spread over a portion of eluting solvent. Therefore, the concentration of the instantaneous eluting slices is quite small, and the $2A_{2}c$ term can be safely neglected. Considering these two situations, Equation (2) can be rewritten as

(6) $$\frac{Kc}{\Delta R}=\frac{1}{M}\left(1+\frac{q^{2}R_{g}^{2}}{3}\right)$$

A further simplification occurs when the $qR_{g}$ product is quite small so that $q^{2}R_{g}^{2}/3<<1$ and, therefore,

(7) $$\frac{Kc}{\Delta R}=\frac{1}{M}$$

This happens if the scattering angle (θ) is sufficiently small and, therefore, the scattering variable (*q*) is also small. This so-called low-angle approximation (LA) may also hold for an arbitrary θ if the solvent molecules are sufficiently small, such that their radius of gyration is much smaller than the light wavelength, $R_{g}/\lambda <<1$. In any case, it will be referred to as the LA approximation.

In addition to conformational (equilibrium) properties extracted from radiation scattering, another set of relevant properties are related to the hydrodynamic (non-equilibrium) behavior of the solution. Such is the case of the intrinsic viscosity, [η], which expresses the increase in the solution viscosity η due to the solute, over the value for the pure solvent $\eta_{0}$. It is defined as the relative viscosity increment, the so-called specific viscosity, $\eta_{sp}$, per unit of solute concentration in the limit of an extremely diluted solution:

(8) $$\left[\eta \right]\equiv \lim_{c\to 0}\frac{\eta_{sp}}{c}=\lim_{c\to 0}\frac{\eta -\eta_{0}}{\eta_{0}c}$$

where

(9) $$\eta_{sp}\equiv \frac{\eta -\eta_{0}}{\eta_{0}}=\eta_{r}-1$$

$\eta_{sp}$ is the specific viscosity and $\eta_{r}\equiv \eta /\eta_{0}$ is the relative viscosity. As indicated above, the concentration of the instantaneously eluting solution is remarkably small in SEC, so that the limit in Equation (8) holds for the observed numerical values.

## 3. Multiple Detection in Size Exclusion

The chromatographic setup should include, at least, a primary detector that simply monitors the concentration $c_{j}$. In some instruments, such a detector is a differential refractometer that monitors the difference in the refractive index, ${\left(\Delta n\right)}_{j}=n_{solution,j}-n_{solvent}$. The signal detected for the *j*-th fraction, obviously adjusted to zero baseline—let us call it ${\left(RI\right)}_{j}$—would be proportional to ${\left(\Delta n\right)}_{j}$, as given by Equation (1)—that is, ${\left(RI\right)}_{j}=Q_{RI}{\left(\Delta n\right)}_{j}$—such that

(10) $${\left(RI\right)}_{j}=Q_{RI}{\left(\frac{dn}{dc}\right)}_{j}c_{j}$$

where $Q_{RI}$ is a constant determined by the instrumental setup. In the usual applications of SEC, the analyte is a single polymer so that the refractive index increment ${\left(dn/dc\right)}_{j}$ is a parameter independent of $c_{j}$ for a sufficiently dilute solution, and can be simply designated as dn/dc.

The light-scattering detectors give a signal which is proportional to the intensity of scattered light, $I=I_{solvent}+\Delta I$. When the eluent is solely solvent, the baseline of the LS detectors would correspond to the solvent contribution, $I_{solvent}$. If the baseline is discounted, the adjusted signal, ${\left(LS\right)}_{j}$, would be directly proportional to the solute contribution ΔI (or, equivalently, ΔR), which, according to Equations (3), (4), and (6), is given by

(11) $$\Delta I=I_{0}r^{-2}\frac{4\pi^{2}}{N_{A}\lambda_{0}^{4}}n_{1}^{2}{\left(\frac{dn}{dc}\right)}^{2}c_{j}M_{j}{\left(1+\frac{q^{2}R_{g,j}^{2}}{3}\right)}^{-1}$$

and the signal can be expressed as

(12) $${\left(LS\right)}_{j}=Q_{LS}n_{1}^{2}{\left(\frac{dn}{dc}\right)}^{2}c_{j}M_{j}{\left(1+\frac{q^{2}R_{g,j}^{2}}{3}\right)}^{-1}$$

where $Q_{LS}$ is an instrumental constant, which involves the factor relating the detector signal (mV) to the intensity of the detected light, as well as the values of *r*, $I_{0}$, and $\lambda_{0}$.

The md-SEC instruments usually include multi-angle LS detection. The simplest setup comprises two detectors, with one of them measuring at a very low angle (e.g., 7°), such that the LA approximation holds and $1+q_{\theta =7}^{2}R_{g,j}^{2}/3\approx 1$ in Equation (12) can be neglected. Therefore, the adjusted signal from such a low-angle detector would be

(13) $${\left(LALS\right)}_{j}=Q_{LALS}n_{1}^{2}{\left(\frac{dn}{dc}\right)}^{2}c_{j}M_{j}$$

Then, the SEC setup includes at least another LS detector at a higher angle, for example, 90° in right-angle light scattering (RALS), for which

(14) $${\left(RALS\right)}_{j}=Q_{RALS}n_{1}^{2}{\left(\frac{dn}{dc}\right)}^{2}c_{j}M_{j}{\left(1+\frac{q_{\theta =90}^{2}R_{g,j}^{2}}{3}\right)}^{-1}$$

We recall that, even for larger angles, the LA approximation may hold for moderately sized solutes. Table 1 presents a list of typical values of the angle dependence of the term $1+q^{2}R_{g}^{2}/3$ for several common yet significant polymers and biomacromolecules, such that the adequacy of the low-angle and right-angle approximations can be shown in various instances. It seems clear that, for most cases—even for quite large solute molecules—the low-angle, angle-independent approximation in Equation (13) is valid. For the non-zero, right-angle scattering detector, Equation (14) can be safely used; even the LA approximation would be valid for moderately sized solutes.

Modern md-SEC setups usually include a detector which is sensitive to the solution viscosity η. In the viscosity detector, the solution and solvent flow through an array of capillaries and their viscosities are related to hydraulic pressures. The quantity being detected varies among different instrument designs. In the Haney instrument [24,25,26,27], the signals from two detectors—namely, absolute (IP) and differential (DP) pressures—are measured. Their combination enables the determination of the specific viscosity of the fraction that is instantaneously eluting, $\eta_{sp,j}$, which is given by the ratio

(15) $$\eta_{sp,j}=\frac{4{\left(DP\right)}_{j}}{{\left(IP\right)}_{j}}$$

From this measurement, the intrinsic viscosity [η] is immediately available. As indicated above, the fractionated solute is remarkably diluted, so the infinite dilution limit in Equation (8) is approximately satisfied and an acceptable approximation for the intrinsic viscosity is

(16) $${\left[\eta \right]}_{j}=\eta_{sp,j}/c_{j}$$

Even for moderately dilute solution, [η] can be determined very accurately using the Solomon–Ciuta equation [28,29,30]:

(17) $${\left[\eta \right]}_{j}=\frac{{\left[2\left(\eta_{sp,j}-\ln\left(1+\eta_{sp,j}\right)\right)\right]}^{1/2}}{c_{j}}$$

A simple numerical procedure can be carried out to extract $\eta_{sp}$ from Equation (17).

### 3.1. Analysis of Data from Concentration Detectors

As the concentration detector monitors the amount of solute in the fraction eluted between ${\left(V_{el}\right)}_{j}$ and ${\left(V_{el}\right)}_{j}+\delta V_{el}$, the conservation of mass is expressed by equalizing the integral over the slice masses to the total mass injected (i.e., to the product of the volume of the injected sample $V_{in}$ and its concentration $c_{in}$).

(18) $$c_{in}V_{in}=\int cdV_{el}={\left[Q_{RI}\left(dn/dc\right)\right]}^{-1}\int \left(RI\right)dV_{el}$$

Furthermore, replacing the integral by a discrete sum over fractions of volume $\delta V_{el}$,

(19) $$c_{in}V_{in}=\sum_{j}c_{j}\delta {\left(V_{el}\right)}_{j}={\left[Q_{RI}\left(dn/dc\right)\right]}^{-1}\sum_{j}{\left(RI\right)}_{j}\delta {\left(V_{el}\right)}_{j}$$

From Equations (18) and (19), the weight fraction $w_{j}$ (i.e., the fraction of the mass eluting in slice *j*, equal to $c_{j}\delta V_{j}$, to the total mass in the sample) can be determined as

(20) $$w_{j}\equiv \frac{c_{j}\delta {\left(V_{el}\right)}_{j}}{\sum_{j}c_{j}\delta {\left(V_{el}\right)}_{j}}=\frac{{\left(RI\right)}_{j}\delta {\left(V_{el}\right)}_{j}}{\sum_{j}{\left(RI\right)}_{j}\delta {\left(V_{el}\right)}_{j}}$$

Thus, the RI trace directly provides a mass distribution of the sample as a function of the elution volume, $w(V_{el})$.

It also follows immediately that

(21) $$c_{j}=\frac{c_{in}V_{in}{\left(RI\right)}_{j}}{\sum_{j}{\left(RI\right)}_{j}\delta {\left(V_{el}\right)}_{j}}$$

such that the concentration of the successively eluting fractions can be evaluated from the RI trace, knowing the injection data $c_{in}$ and $V_{in}$.

Obtaining properties of a problem sample requires the calibration of the detector (i.e., determination of the $Q_{RI}$ constant). This is performed by means of a previous chromatogram with a standard sample whose refractive index increment ${\left(dn/dc\right)}_{s}$ is known. From Equation (19),

(22) $$Q_{RI}={\left[c_{in}V_{in}{\left(dn/dc\right)}_{s}\right]}^{-1}\sum_{j}{\left(RI\right)}_{j}\delta {\left(V_{el}\right)}_{j}$$

Then, for a problem sample, the concentration of the eluting solution can also be obtained as

(23) $$c_{j}=\frac{{\left(RI\right)}_{j}}{Q_{RI}{\left(dn/dc\right)}_{p}}$$

which requires the knowledge of the refractive index increment of the problem sample, ${\left(dn/dc\right)}_{p}$. Nonetheless, if this were unknown, it can be determined if the volume and concentration of the injected sample are known with adequate precision, since the mass conservation Equation (19) yields

(24) $${\left(\frac{dn}{dc}\right)}_{p}=\frac{\sum_{j}{\left(RI\right)}_{j}\delta {\left(V_{el}\right)}_{j}}{Q_{RI}c_{in}V_{in}}$$

The value of ${\left(dn/dc\right)}_{p}$ will be required for the analysis of the traces of the light scattering signals, as described below.

### 3.2. Analysis of Data from Light-Scattering Detectors

The analysis of LS data was performed in terms of Equations (13) and (14). Again, this requires the calibration of the LS detectors from measurements of a standard for which, in addition to ${\left(dn/dc\right)}_{s}$, we should have information on the molecular weight. Note that, if the solutes were absolutely monodisperse, the signal-to-concentration ratio ${\left(LS\right)}_{j}/c_{j}$ should be a constant at any angle. For the calibration, the weight-averaged molecular weight of the standard, $M_{w}$, suffices. Accepting that Equation (13) is valid, it turns out that the weight average of the ${\left(LALS\right)}_{j}/c_{j}$ ratio, given by

(25) $${\langle \frac{{\left(LALS\right)}_{j}}{c_{j}}\rangle }_{w}\equiv \sum_{j}w_{j}\frac{{\left(LALS\right)}_{j}}{c_{j}}=Q_{LALS}n_{1}^{2}{\left(dn/dc\right)}_{s}^{2}\sum_{j}w_{j}M_{j}$$

contains the weight-averaged molecular weight, $M_{w}=\sum_{j}w_{j}M_{j}$. Therefore, the detector constant can be obtained as

(26) $$Q_{LALS}=\frac{1}{n_{1}^{2}{\left(dn/dc\right)}_{s}^{2}M_{w}}{\langle \frac{{\left(LALS\right)}_{j}}{c_{j}}\rangle }_{w}$$

or

(27) $$Q_{LALS}=\frac{1}{n_{1}^{2}{\left(dn/dc\right)}_{s}^{2}M_{w}}\sum_{j}w_{j}\frac{{\left(LALS\right)}_{j}}{c_{j}}$$

In the general case of RALS, the determination of $Q_{RALS}$ from molecular weights is more involved due to the influence of the radius of gyration. If the LA approximation is applicable at any angle, equations similar to (25), (26), and (27) would give $Q_{RALS}$ from the ${\left(RALS\right)}_{j}$ signals. Thus,

(28) $$Q_{RALS}=\frac{1}{n_{1}^{2}{\left(dn/dc\right)}_{s}^{2}M_{w}}\sum_{j}w_{j}\frac{{\left(RALS\right)}_{j}}{c_{j}}$$

In the particular case of a monodisperse polymer sample of molecular weight *M*, the polymer eluting at any *j*-th slice is always the same, such that ${\left(LALS\right)}_{j}/c_{j}$ and ${\left(RALS\right)}_{j}/c_{j}$ in the LA approximation (Equations (13) and (14)) are constant, and the previous equations reduce to

(29) $$Q_{LALS}=\frac{1}{n_{1}^{2}{\left(dn/dc\right)}_{s}^{2}M}\frac{{\left(LALS\right)}_{j}}{c_{j}}$$

(30) $$Q_{RALS}=\frac{1}{n_{1}^{2}{\left(dn/dc\right)}_{s}^{2}M}\frac{{\left(RALS\right)}_{j}}{c_{j}}$$

In the analysis for a problem sample—based on the assumption, again, that Equation (13) holds—the molecular weight of the solute fractions can be obtained as follows:

(31) $$M_{j}=\frac{{\left(LALS\right)}_{j}}{Q_{LALS}n_{1}^{2}{\left(dn/dc\right)}_{p}^{2}c_{j}}$$

For RALS in the LA approximation,

(32) $$Q_{RALS}=\frac{1}{n_{1}^{2}{\left(dn/dc\right)}_{s}^{2}M_{w}}\sum_{j}w_{j}\frac{{\left(RALS\right)}_{j}}{c_{j}}$$

and, conversely, the molecular weight of the eluting polymer would be

(33) $$M_{j}=\frac{{\left(RALS\right)}_{j}}{Q_{RALS}n_{1}^{2}{\left(dn/dc\right)}_{p}^{2}c_{j}}$$

We note that the LALS–RALS ratio

(34) $$\frac{{\left(RALS\right)}_{j}}{{\left(LALS\right)}_{j}}=\frac{Q_{RALS}}{Q_{LALS}}\frac{1}{1+q_{\theta =90}^{2}R_{g,j}^{2}/3}$$

would be constant, equal to the ratio $Q_{RALS}/Q_{LALS}$, if the low-angle approximation would also hold at higher angles. Otherwise, from the ratio of the two LS signals, one could determine the radius of gyration, $R_{g,j}$, of the solute fractions.

## 4. Analysis of SEC Data

### 4.1. Analytical Tools: ReadSECRaw, SECcal and SECanal

#### 4.1.1. ReadSECRaw 1.0

Based on the above-described theoretical formalism, we have devised a first (1.0) version of simple computational tools for the analysis of sized-exclusion chromatograms with multiple (refractive index, two-angle LS, and viscosity) detection approaches. SECcal 1.0 is used for the previous calibration of the detectors, and SECanal 1.0 is in charge of determining molecular weight distributions and analyzing the molecular weight dependence of the properties.

The primary input for both programs is in user-supplied fixed-format files containing the raw chromatographic data. One file, RawSignals.txt, contains just a list of the raw values of the refractometer, scattering, and viscosity detector signals: ${\left(RI\right)}_{j}$, ${\left(LALS\right)}_{j}$, ${\left(RALS\right)}_{j}$, ${\left(IVDP\right)}_{j}$, and ${\left(IVIP\right)}_{j}$ at each elution volume, ${\left(V_{el}\right)}_{j}$. Users would construct this standard chromatographic file from their specific data-acquisition setup. Another file, InitData.txt, contains some basic data, namely, the injection volume $V_{in}$, the concentration of the injected sample $c_{in}$, the refractive index increment dn/dc of the solute, and the refractive index of the solvent $n_{1}$.

**Figure 2:**
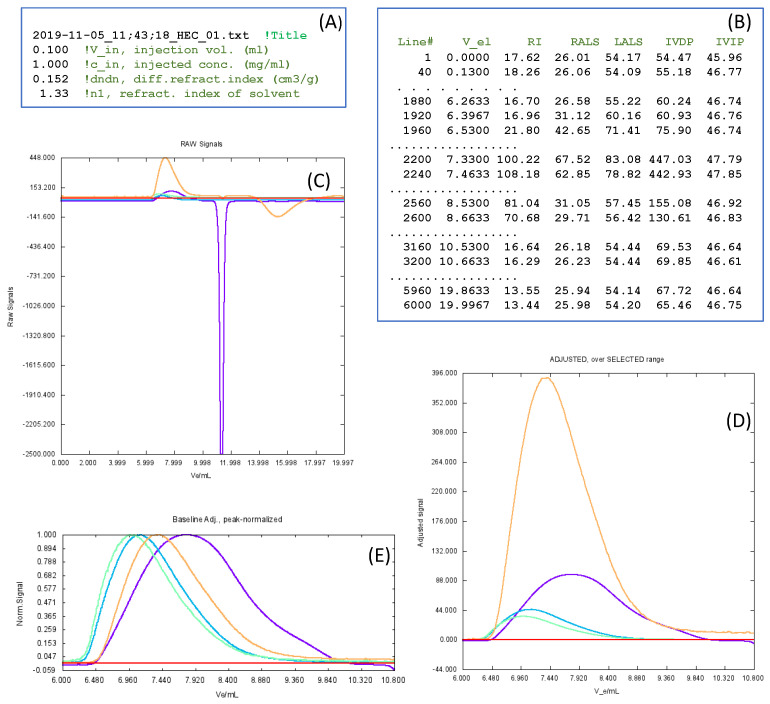
Some details about the execution of ReadSECRaw 1.0. (**A**,**B**) Examples of the two input files: InitData.txt and ReadSECRaw 1.0. Text in green corresponds to labels that have been added here, but do not belong to the real files. In RawSignals.txt, only a few selected lines are shown. (**C**) Plot of the raw chromatograms corresponding to each detector, with the colors being as follows: RI, violet; LALS, dark green; RALS, light green; IVDP, brown; IVIP, red. Seeing this plot, the user will determine the limits of the range of elution volume, $V_{el}$, which contains the peaks. In this example, it was $V_{el}$ = 6.0–10.8 mL. This way, instrumental artefacts and other features foreign to the macromolecular solute—such as the strong overshoot seen in this case—can be eliminated. Then, the program selects this range, determines the baseline for each detector and calculates them, displays the zero-baseline-corrected chromatograms—plot (**D**)—and stores the values in the file AdjustedSignalsSelected. Finally, the program constructs and visualizes a plot, (**E**), of chromatograms that are further normalized to the unit peak height. (**C**–**E**) are screenshots of plots displayed during program execution.

Then, our program ReadSECRaw 1.0 is responsible for the following tasks: (i) estimating the baseline of each of the detectors and adjusting each detector record to a zero baseline; (ii) establishing a range of the elution volume where all the significant information (peaks) is contained; (iii) determining the maximum peak values and making a plot of the superimposed chromatograms normalized to unit peak height; (iv) calculating the specific viscosity, $\eta_{sp}$, from the adjusted values of the two viscosity detectors (Equation (15)); and (v) writing a file named AdjustedData.txt, containing a list of the baseline-adjusted values of the detector signals for each value of the elution volume—that is, ${\left(V_{el}\right)}_{j}$, ${\left(AdRI\right)}_{j}$, ${\left(AdRALS\right)}_{j}$, ${\left(AdLALS\right)}_{j}$, and $\eta_{sp}$. This file is the input of our calibration program, SECcal 1.0, as well as our analysis program, SECanal 1.0. Figure 2 presents some details of the files and plots, which are displayed by ReadSECRaw 1.0 during program execution.

#### 4.1.2. SECcal 1.0

The required previous calibration of the detectors is carried out using a standard (i.e., a polymer with a known molecular weight distribution or averages).

SECcal 1.0 first treats the RI data: With the integral (as a discrete sum) of the RI signal, the constant of the RI detector, $Q_{RI}$ as defined in Equation (10), is obtained with basis on Equation (22) using the values of $V_{in}$ and $c_{in}$.

The calibration of LS detectors is simpler if the polymer standard has a moderate size, such that the LA approximation applies not only for LALS, but for RALS as well. Equations (27) and (28) could be applied to average over the whole peak to extract the $Q_{LALS}$ and $Q_{RALS}$ from the weight-averaged molecular mass, $M_{w}$. For either LALS or RALS, the weight averages ${\langle {\left(LS\right)}_{j}/c_{j}\rangle }_{w}$—required in Equations (26) and (27)—are evaluated from the ${\left(LS\right)}_{j}$ registers with the weight fractions obtained from the ${\left(RI\right)}_{j}$ registers using Equations (20) and (21).

Furthermore, if the standard is a nearly monodisperse polymer of molecular weight *M*, then detector constants $Q_{LALS}$ and $Q_{RALS}$ can be determined from Equations (29) and (30), using the values of ${\left(LALS\right)}_{j}$ and ${\left(RALS\right)}_{j}$, along with the concentration, $c_{j}$, of any particular fraction in the chromatogram (concentrations $c_{j}$ are directly evaluated from the $RI_{j}$ signal according to Equation (21)). The *j*-th fraction can be that which maximizes the LS signal (i.e., the top of the peak), which maximizes the signal-to-noise ratio.

#### 4.1.3. SECanal 1.0

Once the detector constants have been determined, they are employed in the SECanal 1.0 program to analyze the chromatograms of problem samples. The weight fraction for each slice, $w_{j}$, can be directly evaluated from the ${\left(RI\right)}_{j}$ signals using Equation (20). Having the value of $c_{in}V_{in}$, the concentrations are just $c_{j}$ = $c_{in}V_{in}w_{j}$. If the refractive index increment of the sample ${\left(dn/dc\right)}_{p}$ is unknown, it can be evaluated using the constant of the RI detector by means of Equation (24).

Alternatively, knowing the ${\left(dn/dc\right)}_{p}$ of the sample, the product $c_{in}V_{in}$ can be evaluated from Equation (19), and then proceed to obtain the $c_{j}$ values as mentioned. If both data points $c_{in}V_{in}$ and ${\left(dn/dc\right)}_{p}$ are available, then the $c_{j}$ values can be carried out with both procedures to check their concordance.

With the series of values of the LS detectors for each slice, SECanal 1.0 obtains the value of the molecular weight, $M_{j}$, both from ${\left(LALS\right)}_{j}$ (Equation (31)) and from ${\left(RALS\right)}_{j}$ (Equation (33)). If the LA approximation holds for both signals, then the two values should coincide, although differences due to instrumental effects may show up. Next, SECanal 1.0 evaluates the intrinsic viscosity, ${\left[\eta \right]}_{j}$, from the combination of signals from the viscosity detectors and concentration, according to Equation (16) (recall that the viscosity analysis does not require previous calibration).

Then, SECanal 1.0 proceeds, combining all this resulting information in several ways. Combining the $M_{j}$ vs. ${\left(V_{el}\right)}_{j}$ values with the $w_{j}$ vs. ${\left(V_{el}\right)}_{j}$ values, the resulting series of $w_{j}$ vs. $M_{j}$ values provides an essential result in SEC, namely the molecular mass distribution. SECanal 1.0 also provides averages of molecular weight. The weight average is simply

(35) $$M_{w}=\sum_{j}w_{j}M_{j}$$

The number fractions are evaluated as

(36) $$x_{j}=\frac{w_{j}/M_{j}}{\sum_{j}w_{j}/M_{j}}$$

and this enables the determination of other averages, for instance, the number and z averages:

(37) $$M_{n}=\sum_{j}x_{j}M_{j}$$

(38) $$M_{z}=\frac{\sum x_{j}M_{j}^{3}}{\sum x_{j}M_{j}^{2}}$$

Furthermore, SECanal 1.0 combines the $M_{j}$ vs. ${\left(V_{el}\right)}_{j}$ with the ${\left[\eta \right]}_{j}$ vs. ${\left(V_{el}\right)}_{j}$. The resulting series of ${\left[\eta \right]}_{j}$ vs. $M_{j}$ describes the molecular weight dependence of the intrinsic viscosity, which is an essential indicator of the polymer conformation. The intrinsic viscosity of the unfractionated bulk sample is also evaluated. As the contribution of the components of a heterogeneous polymer to the viscosity increments are additive and proportional to concentrations, the bulk intrinsic viscosity is given by

(39) $${\left[\eta \right]}_{bulk}=\sum_{j}w_{j}{\left[\eta \right]}_{j}$$

Although these results may be of secondary interest, it could be useful to compare them with those from a separate measurement using conventional viscosimetry, as a way of checking the SEC determinations.

#### 4.1.4. Workflow

**Figure 3:**
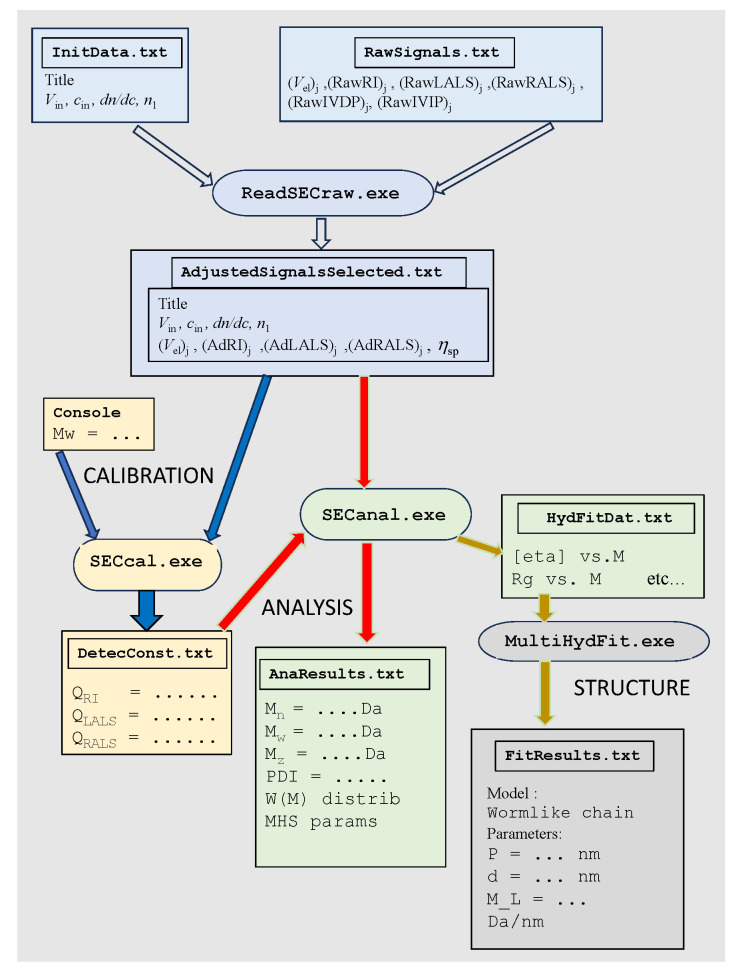
Workflow of the computer programs in SECtools. Blue blocks are those of data pre-processing treatment, common to calibration (orange) and analysis (green). Gray blocks are those of post-processing with MultyHydFit.

Figure 3 presents a workflow indicating in different colours numeric and executable files in the various stages of data analysis. The values indicated as (AdRI)_j_, (AdLALS)_j_, and (AdRALS)_j_ are the baseline-adjusted detector signals which are, obviously, those used in the analysis. The diagram includes the optional post-processing with MultyHydFit, as described in a later section.

## 5. Experimental Results

Here, we present a series of results on real chromatographic experimental data obtained with our chromatograph for several polymer solutions and present the work flow for their analysis using our programs of our SECtools suite, namely, ReadSECRaw 1.0, SECcal 1.0, and SECanal 1.0.

### 5.1. Instrumentation and Materials

The chromatograph used in our experimental measurements was the Viscotek GPCmax VE-2001 (purchased from Malvern Instruments Ltd., Malvern, UK), coupled to the triple detector array TDA 305, both from Malvern-Viscotek. The detector array includes the following: (a) a differential refractometer measuring the refractive index increment, δn; (b) light-scattering detection at two angles—a right angle, RALS, at 90°, and a low angle, LALS, at 7°, with 3 mW laser diode, with the light source being a laser with a wavelength of $\lambda_{0}=$ 670 nm; and (c) a viscosity detector consisting of four capillaries arranged as a differential Wheatstone bridge configuration [24,25]. The setup was equipped with a guard pre-column and one or two size-separating columns, namely, one A4000 column from Malvern-Viscotek and one PL-aquagel-OH-40-8 μm column from Agilent Technologies (Santa Clara, CA, USA), serially connected to the first one. The integrated detectors and columns were fully temperature-controlled, with the working temperature being 303 K. The flow rate was 0.7 mL/min in all cases and the injection volume was 100 μL.

Instrument control and data acquisition were performed using the Malvern-Viscotek OmniSEC 4.7.0 software. This is a comprehensive software, which additionally carries out a sophisticated protocol for data analysis; however, for the purpose of collecting the raw data to be processed with SECtools programs, OmniSEC 4.7.0 was—in principle—employed just for running the chromatogram and gathering the raw detector signals (mV).

The primary file with the raw experimental data used in our SECtools, named RawData.txt, will first contain the injection data, $V_{in}$ and $c_{in}$, and the refractive index data, dn/dc and $n_{1}$, followed by a tabular list of values of the elution volume, ${\left(V_{el}\right)}_{j}$, and the raw signals (mV) of the five detectors, ${\left(RI\right)}_{j}$, ${\left(LALS\right)}_{j}$, ${\left(RALS\right)}_{j}$, ${\left(IVDP\right)}_{j}$, and ${\left(IVIP\right)}_{j}$, at the *j*-slices of the chromatogram.

In order to obtain the data for RawData.txt from the OmniSec software, as mentioned above, we wrote an ancillary program, ReadSECRaw 1.0, which can extract the signal data of the TDA 305 module of the Viscotek GPCmax VE-2001. Hopefully, users of other SEC instrumentation will be able to do so for their own interface to SECtools.

### 5.2. Calibration with Pullulan Standard

The Pullulan standard from Malvern-Viscotek (PolyCAL, referenced PUL-71K, Pul71 in our notation) is practically monodisperse with $M_{w}$ = 70,768 Da. This was used to calibrate the instrument to obtain the instrument constants $Q_{RI}$, $Q_{LALS}$, and $Q_{RALS}$ (Equations (22), (27), and (32)) by means of SECcal 1.0. The sample concentration was 1 mg/mL.

The chromatograms recorded by OmniSec 4.7.0 were transferred by our ReadSECRaw 1.0 program to file RawData.txt, which is the standard format employed in SECtools. This file was then processed by ReadSECRaw 1.0, which—as indicated above—is in charge of building the input of our calibration program, SECcal 1.0.

**Figure 4:**
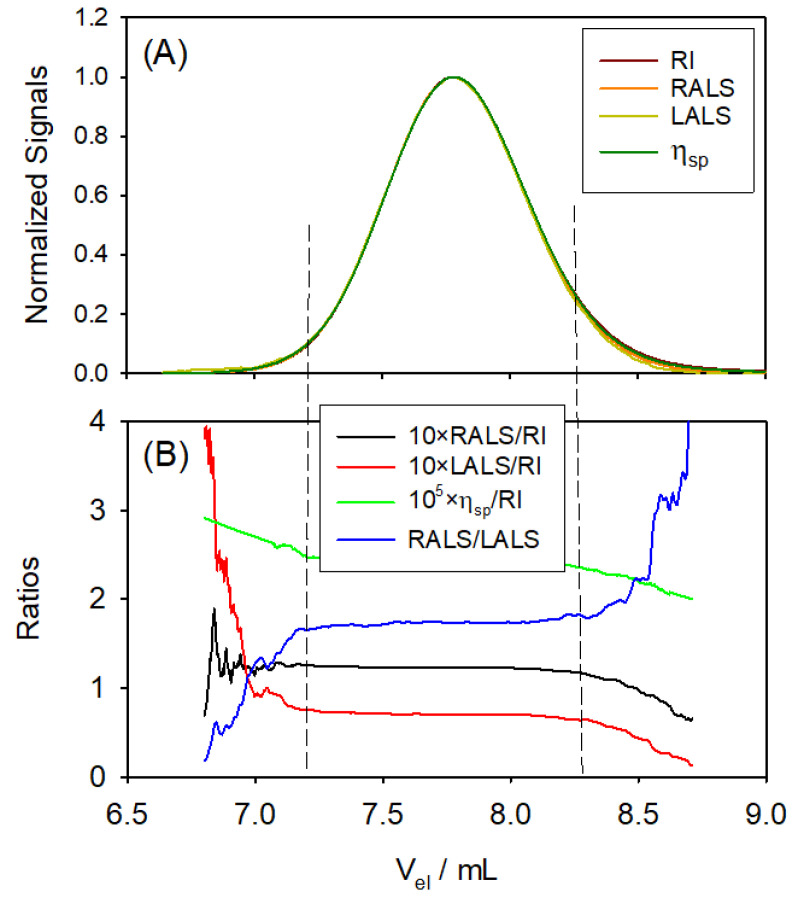
Experimental results for Pullulan Standard 71 kDa (Pul71). (**A**) Resulting multi-detection chromatograms (normalized signals vs. elution volume) of the standard Pul71. (**B**) Signal ratios vs. elution volume. The fluctuations at the tails are a consequence of the very low values of the signals—blurred by detector noises—when concentration is very low.

Figure 4A shows the resulting multi-detection chromatograms (signals vs. elution volume, ${\left(V_{el}\right)}_{j}$) of the standard Pul71. As can be appreciated, they superimpose perfectly. The polymer elutes over a range of about 2 mL (approximate peak width). An immediate analysis can be made from combinations of detector signals. According to Equations (13) and (14), the LS signals are proportional to the concentration, $c_{j}$, of the eluting polymer; therefore, their ratios to ${\left(RI\right)}_{j}$ are as follows:

(40) $$\frac{{\left(LALS\right)}_{j}}{{\left(RI\right)}_{j}}=\frac{Q_{LALS}}{Q_{RI}}n_{1}^{2}\left(\frac{dn}{dc}\right)c_{j}M_{j}$$

(41) $$\frac{{\left(RALS\right)}_{j}}{{\left(RI\right)}_{j}}=\frac{Q_{RALS}}{Q_{RI}}n_{1}^{2}\left(\frac{dn}{dc}\right)c_{j}M_{j}{\left(1+\frac{q_{\theta =90}^{2}R_{g,j}^{2}}{3}\right)}^{-1}$$

The concentration dependence is factored and would depend on $M_{j}$ and, eventually (if the polymer is large, which is not the case for Pul71) on its size, $R_{g,j}$. Likewise, from Equation (16), $\eta_{sp,j}$ is proportional to $c_{j}$ and the intrinsic viscosity ${\left[\eta \right]}_{j}$, so that the ratio $\eta_{sp,j}/{\left(RI\right)}_{j}$ depends only on ${\left[\eta \right]}_{j}$.

In Figure 4B, these signal ratios are plotted vs. $V_{el}$. The strong fluctuations at the tails of the peak are a simple consequence of the very low values of the signals—which are blurred by detector noises—when the concentration is very low. In the peak region of eluted volume, we note that the ratios are constant, as $M_{j}$ is obviously the same for all the eluting slices; the polymer is, certainly, monodisperse. Furthermore, the signals corresponding to the various properties show the same trend and reach their maximum peak values at the same $V_{el}$, as seen in Figure 4B.

After these inspections, the detector constants are determined using SECcal 1.0. From the given values of $c_{in}$, $V_{in}$, and dn/dc, contained in the chromatogram file, SECcal 1.0 uses Equation (22) to give the constant of the RI detector, given in Table 2, and Equation (23) enables the calculation of $c_{j}$ for every eluting slice. SECcal 1.0 then proceeds to evaluate the LS detector constants. This is performed first from the peak values; the slice that elutes at the peak has the maximum concentration for which $c_{j}=1.39\times 10^{-4}$ g/mL. Then, Equation (29) is used to obtain $Q_{LALS}$. With a solute of moderate size, the $R_{g}$-dependent term can be ignored, with $1+q_{\theta =90}^{2}R_{g,j}^{2}/3\approx 1$. For Pullulan with M=71 kDa, the radius of gyration should be about 8.9 nm [15], and $q_{\theta =90}^{2}R_{g,j}^{2}/3=0.008$, which certainly fulfils this condition. Then, the constant of the RALS detector can be determined from Equation (30). SECcal 1.0 also obtains the constants of the LS detectors, obtaining weight averages by integration over the whole chromatogram, thus evaluating $Q_{LALS}$ from Equation (26) and $Q_{RALS}$ from Equation (28). The numerical results are presented in Table 2.

### 5.3. Monodisperse and Paucidisperse Samples

In order to safely check the performance of our data-treatment programs, we proceeded to apply them to experiments carried out with well-characterized samples, containing pure monodisperse polymers or mixtures of them.

In a somehow trivial but illustrative exercise, we consider Pul71 as an unknown sample and process its data files with SECanal 1.0. The results are displayed in Table 3. The excellent agreement with the expected molecular weight was to be expected, as it is the very same sample used for calibration. The merit is, simply, that this confirms the proper functioning of SECanal 1.0. Next, we run three other monodisperse Pullulan samples—namely, Pul48, Pul113, and Pul210 in our notation—purchased from Sigma-Aldrich, Buchs, Switzerland (Fluka, PSS—Pullulan Standard Set (Product number 96351)). The numerical data obtained for the molecular weight are listed in Table 3. The agreement with the nominal values from the standards is most rewarding.

**Figure 5:**
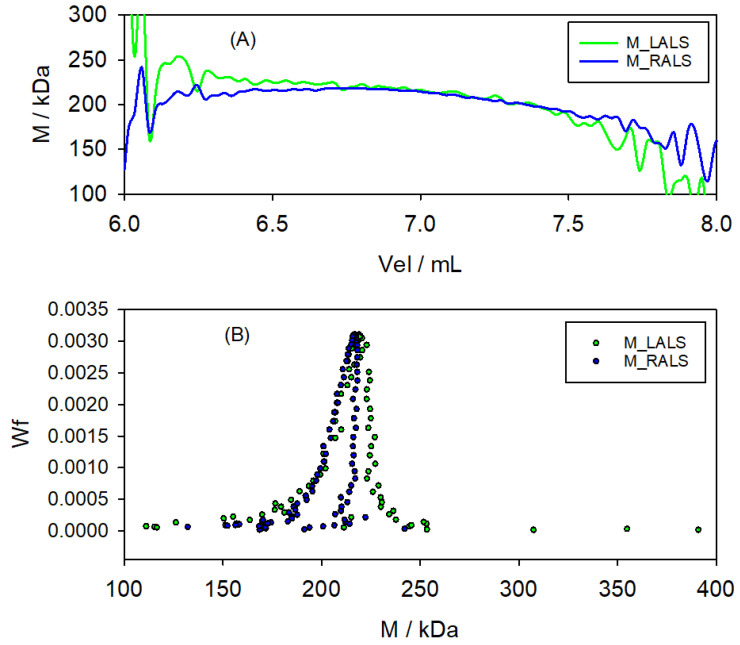
Some results for the Pul210 sample. (**A**) *M* determined from the LALS and RALS detection, for each slice over the range of the chromatographic peak. (**B**) Molecular weight distribution (weight fraction) obtained from the LALS and RALS detectors. As can be appreciated, the molecular weights for each slice within the peak region are practically identical and very close to the nominal one.

Some details of the analysis for sample Pul210 are shown in Figure 5, which shows how the molecular weights for each slice within the peak region, ${\left(M_{LALS}\right)}_{j}$ from Equation (31) and ${\left(M_{RALS}\right)}_{j}$ from Equation (33), are practically identical, and very close to the nominal one. The molecular weight distribution, shown in the plot of $w_{j}$ vs. ${\left(M_{LALS}\right)}_{j}$ and vs. ${\left(M_{RALS}\right)}_{j}$, presents a sharp peak, as expected, at the nominal *M*.

Table 3 also contains the SECanal 1.0 results for the intrinsic viscosity, which can be compared with the literature data. Kasaai [31] has reported a Mark–Houwink–Sakurada equation for Pullulan:

(42) $$\left[\eta \right]\left(mL/g\right)=0.01956M^{0.667}$$

As shown in Table 3, our results for [η] of Pullulan standards are in good agreement with the numerical values obtained from Equation (42) with the nominal *M* of the standards.

A paucidisperse, bicomponent sample—Pul48+805 in our notation—was prepared by mixing two monodisperse Pullulan standards with widely separated molecular weights: 48 kDa and 805 kDa (Fluka, Pullulan Standard Set (Product number 96351), purchased from Sigma-Aldrich, Buchs, Switzerland).

**Figure 6:**
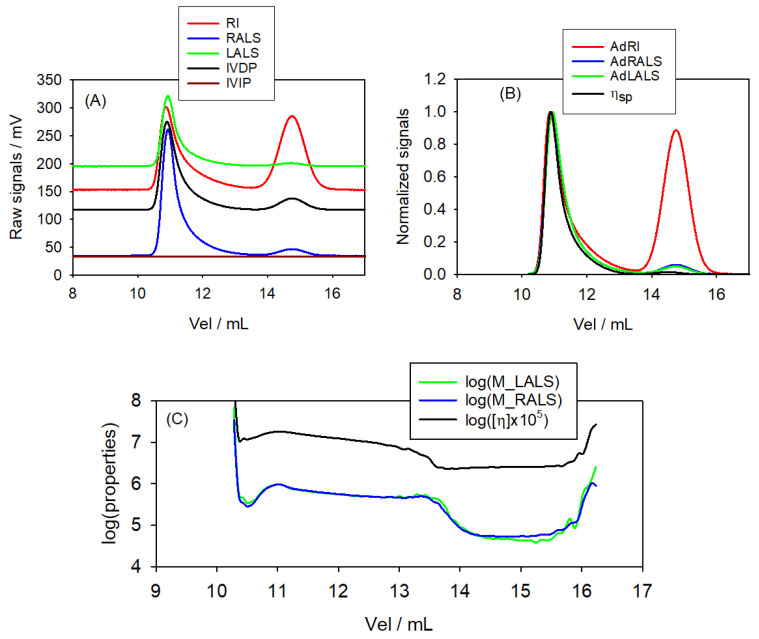
Chromatograms of the Pul48+805 bimodal sample. (**A**) Raw signals from all the detectors. (**B**) Signals adjusted to zero-baseline, and values of the specific viscosity (Equation (15)); all values normalized to unit value for the height of the highest peak. (**C**) Molecular weights, ${\left(M_{LALS}\right)}_{j}$ and ${\left(M_{RALS}\right)}_{j}$, and intrinsic viscosity, [η], calculated at each slice for each ${\left(V_{el}\right)}_{j}$. Plots of log(properties) vs. $V_{el}$ for this bidisperse sample present two plateaus corresponding to each component peak.

The chromatogram displayed in Figure 6 shows two peaks clearly, the one eluting to lower $V_{el}$ corresponding to the higher *M*, and that eluting to higher $V_{el}$ corresponding to the lower *M*.

Plots of properties vs. $V_{el}$ for this bidisperse sample present two regions, each with a plateau region corresponding to the peak of each component. Values of *M* and [η] of each component can be estimated as the values of the slices in each chromatogram, and the values are included in Table 3. These approximate values are in reasonable agreement with the expected ones, thus showing the qualitative and semi-quantitative good performance of our setup for more complex samples.

### 5.4. A Polydisperse Sample: 2-Hydroxyethyl Cellulose

Water-soluble cellulose derivatives are appreciably inexpensive polymers that have a number of quite diverse applications, and are particularly suited to be characterized in solution by md-SEC; 2-hydroxyethyl cellulose (2HEC) is a relevant example [32].

Our sample of 2HEC was purchased from Sigma-Aldrich, Buchs, Switzerland (Product number 434964). The procedure for registering the chromatograms and their analysis with our SECtools programs was the same as for the Pullulan samples.

**Figure 7:**
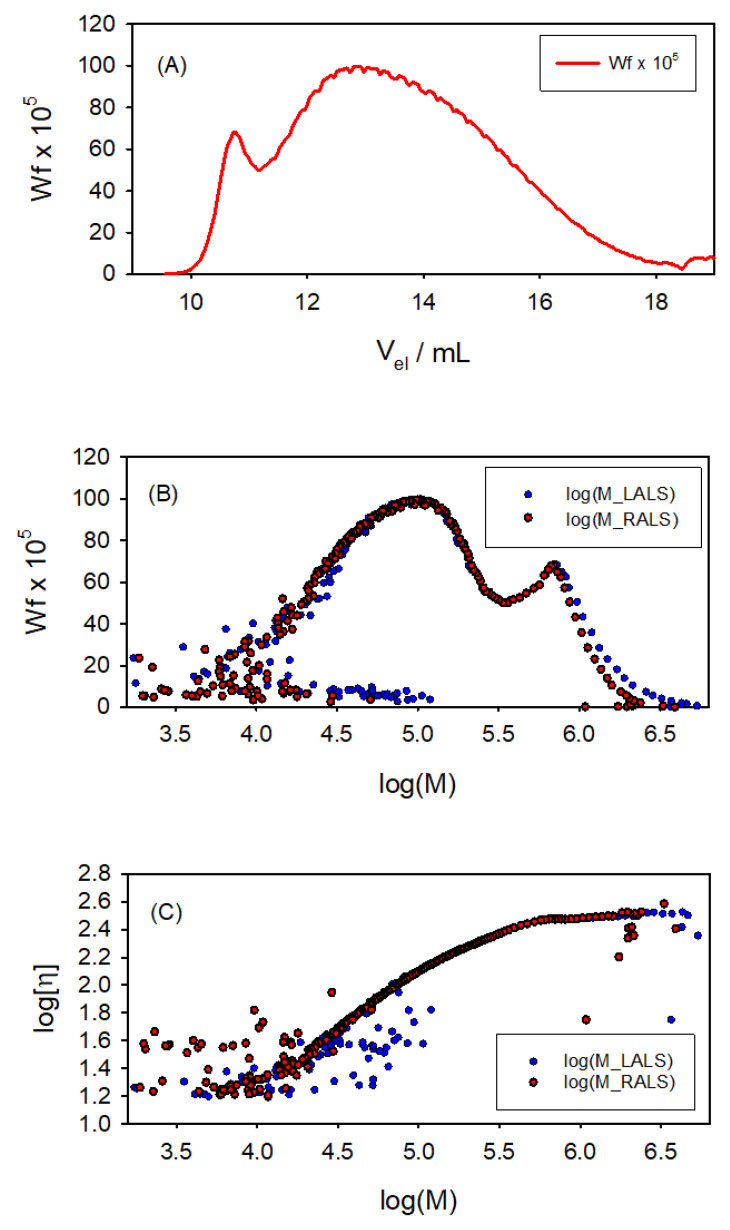
Results for 2-hydroxyethyl cellulose. (**A**) Weight fraction along the chromatographic record. (**B**) Molecular weight distributions from both LALS and RALS detectors. (**C**) Log–log plot of [η] vs. *M*, both LALS and RALS. A somehow bimodal composition is appreciated for this sample, as if it were a mixture of a widely polydisperse component, ranging from $M=10^{3}$ to $M=10^{6}$ Da, and a second component with a peak at about *M*∼$3\times 10^{5}$ Da.

Figure 7 presents some of the results obtained with the SECtools programs. The molecular weight distribution, expressed as weight fraction $w_{f}$, shows an interesting aspect of this sample: a somehow bimodal composition, as if it were a mixture of a majority, widely polydisperse component, with a range of *M* from 10^3^ to 10^6^ Da; and a second component, which appears at $V_{el}=9.8$ mL with a sharper *M* distribution with a peak at about logM∼5.7 (i.e., *M*∼300 kDa). We also obtained the global properties of the sample; namely, the average molecular weights $M_{n}=33$ kDa, $M_{w}=181$ kDa, and $M_{z}=644$ kDa, with a polydispersity index of PDI∼6. The $M_{n}$ and $M_{z}$ results are less reliable than those for $M_{w}$, as those averages are more influenced by the shorter and longer polymer chains, respectively, whose *M* values are affected by instrumental noise. The reason is that they elute at the higher and lower values of $V_{el}$, respectively, at which the signal-to-noise ratio of the detectors is important (as evidenced in Figure 7). Furthermore, the global intrinsic viscosity is found to be [η]= 115 mL/g.

The wide molecular weight distribution in this sample also provides its use to illustrate the applicability of the SECtools to obtain structural information, as described in the next section.

## 6. Structural Determination

In addition to the molecular weight as the primary structural aspect, the dilute-solution properties of macromolecules are an essential source of information on the global structure. The dependence of properties like the intrinsic viscosity, [η], and the radius of gyration, $R_{g}$, on molecular weight, *M*, depends on the macromolecular chain flexibility; therefore, it is informative about whether the macromolecule is rigid or flexible. For rigid macromolecules, that dependence will be related to the shape, and if flexible, the dependence will be informative about intramolecular and solute–solvent interactions. The possibility of determining the [η]–*M* relationship from the md-SEC of polydisperse samples is particularly valuable, as shown in the above examples; this is also the case for the $R_{g}$–*M* relationship if the macromolecule is quite large, and even the ratio $R_{g}/R_{h}$, where $R_{h}$ is the hydrodynamic radius obtained from [η] and *M* [12].

When the solution behavior of the macromolecule can be described by simple models, such as the random-coil (for very long and flexible polymers) or the spherical particle (for some globular proteins), the conformation–property relationships are expressed by simple, usually power-law equations of property vs. molecular weight. However, there are cases for which the theoretical aspects of the model are more complicated, and the extraction of structural parameters from solution property data is more involved. For such cases, we have developed a computer program, MultiHydFit [11,33], which embodies the theory for other models [12], such as short cylindrical particles (short rods and disks) [34] and semi-flexible, Kratky–Porod, worm-like chains (WCs) [35]. The procedure is based on the concept of equivalent radii [11]. The input data are a series of molecular weight values, *M*, and their corresponding property values, *p*, where *p* can be any of the diverse solution properties—including not only [η] and $R_{g}$ but also including scattering-related and hydrodynamic properties like diffusion and sedimentation. MultiHydFit implements a global fitting procedure for obtaining optimal values of the structural parameters which, in the case of the WC model, are the persistence length *P* that indicates the degree of stiffness of the chain, its diameter (thickness), *d*, and the mass per unit of contour length, $M_{L}$.

Within the SECtools package, we include an interface that connects the output of SECanal 1.0 to MultiHydFit. The series of $M_{j}$, ${\left[\eta \right]}_{j}$, and eventually $R_{g,j}$—for the many fractions contained in the many slices of the chromatograms—is transferred to a MultiHydFit input file, and then this program obtains the structural parameters. For polymers like polysaccharides, which may present some degree of chain stiffness, the WC is a most appropriate representation. Then, we have used the results for 2-hydroxyethyl cellulose to obtain the WC parameters, obtaining P=2.4 nm, d=1.6 nm, and $M_{L}=48.8$ Da/nm. The *P* value is not high—it is appreciably smaller than that of other polysaccharides; thus, the cellulose skeleton of the 2HEC derivative is quite flexible. Chain stiffness is the consequence of intramolecular interactions, which, in the case of polyelectrolytes, include electrostatic repulsions. Such is the case for another polysaccharide, alginate, whose stiffness and its corresponding value of *P* depend on ionic strength. In a previous paper, we used a primitive version of SECtools in an md-SEC study to characterize this dependence [36].

## 7. Conclusions

In this work, we intended to present the basic aspects of the physical properties of macromolecules in solution and how they are the foundations of the technique of multi-detection size-exclusion chromatography, md-SEC. These fundamental aspects of md-SEC, which are described here with a tutorial purpose, may either be beyond the scope of textbooks, or may somehow have been overlooked in some monographs that are mainly focused on practical aspects and applications. Therefore, the conceptual and mathematical contents of this review may be valuable to beginners in the field and, as such, even for training and teaching purposes.

We implemented the fundamentals in a series of programs for SEC data analysis, contained in our public domain suite SECtools, with open-source codes. Although the SECtools suite of programs basically has the same functionality as other typical programs for GPC analysis—namely, the determination of the molecular weight distribution, molecular weight averages, and the relationship between intrinsic viscosity and the molecular weight—it provides an interface to MultiHydFit (our program for the structural analysis of macromolecules) and is vendor-independent: the input for the data analysis is simply a list of values of the raw (mV) RI, LALS, RALS, IVDP and IVIP detector signals vs. elution volume, $V_{el}$. In addition, SECtools has an easy-to-use console dialogue interface and displays graphs during runtime for the visualization of intermediate results, allowing a user to fine-tune the region to be analyzed. We note, also, the valuable possibility of reading, on one hand, the Fortran source code of the programs and, on the other hand, the mathematical basis of the code as described in this paper. The performance of SECtools to extract all the possible information from md-SEC with sufficient accuracy was demonstrated in this article.

Classically, as well as at present, the study of the global structure (i.e., the conformation of macromolecules in solution) was based on measurements of solution properties and the characterization of property–*M* dependencies and *M*-dependent property–property relationships. Years ago, this process was performed by carrying out fractional precipitation and, more recently, by preparative chromatography, in order to extract a sufficient number of fractions of sufficiently narrow dispersion, followed by measuring the solution properties of each fraction one after another. With md-SEC, these studies are successfully facilitated. With a single, widely polydisperse sample, a single chromatogram followed by a few steps with appropriate software can produce the different property vs. *M* data needed for that purpose. As mentioned above, the inclusion of MultiHydFit within SECtools provides an efficient way to obtain structural information. One could affirm that polydispersity was a source of difficulty when used for this purpose years ago; however, at present, by means of md-SEC, it can be regarded as an advantage. With a single, sufficiently polydisperse sample, one can measure the *M* dependence of properties such as [η] and $R_{g}$ over a wide range of *M* values. MultiHydFit can be extended to include other structural models; for instance, rod-like cylinders, disk-like cylinders [33,34] and planar-shaped nanoplatelets [37] for rigid particles, or flexible models for flexible chains with different kinds and extents of branching [38,39,40].

Our description is particularized for a simple md-SEC instrumentation consisting of a concentration detector—namely, a differential refractometer—a dual-angle light-scattering detector, and a capillary-bridge differential viscometer. The extension of the theory and the computational tools to cover md-SEC with multi-angle light-scattering (SEC-MALS) is straightforward; indeed, an array of detectors can be considered in the same way as just a pair. The less-used dynamic light-scattering (SEC-DLS) detector provides an alternative or complementary way to include (like the viscosity detector) a hydrodynamic property, the diffusion coefficient, or the Stokes radius in the information gathered from md-SEC (see, for instance, ref. [41]). Even the more sophisticated detection based on small-angle X-ray scattering (SEC-SAXS) may be a complement to LS to measure the radius of gyration, $R_{g}$, and other structural properties of small macromolecules, such as low-molecular-weight polymers, oligomers, proteins, and RNAs [42,43,44,45].

## 8. Computer Programs

The SECtools suite of computer programs (ReadSECRaw 1.0, SECcal 1.0, and SECanal 1.0) will be freely available, including source codes, detailed user guides, and several examples of input and output files, on our website URL: https://leonardo.inf.um.es/macromol.

## Figures and Tables

**Table 1:** Values of the angle-dependent term in Equations (12) and (14) at low-angle and right-angle scattering for several synthetic and biological macromolecules in aqueous media: (1) poly(ethylene oxide); (2) immunoglobulin, IgM.

Scattering Angle, θ				7°	90°
**q/${\mathrm{nm}}^{-1}$ ($\lambda_{0}=632.8$ nm, n=1.33)**				**0.0016**	**0.0187**
**Polymer System**	**M/kDa**	**$R_{g}$/nm**	**Ref.**	** $1+q^{2}R_{g}^{2}/3$ **	** $1+q^{2}R_{g}^{2}/3$ **
Pullulan	100	10.9	[15]	1.0001	1.014
Schizophyllan	620	72.3	[16]	1.004	1.61
Dextran	26.6	47	[17]	1.002	1.26
PEO (1)	20	4.3	[18]	1.00002	1.0021
PEO (1)	600	44.9	[19]	1.002	1.23
Lysozyme	14.3	1.43	[20]	1.000002	1.0002
IgM (2)	950	12.1	[21]	1.0001	1.017
DNA fragment (432 base pairs)	281	34.6	[22]	1.001	1.16
5S RNA	44.5	3.27	[23]	1.000009	1.0012

**Table 2:** Constant detectors for RI, LALS, and RALS, determined by calibration with Pullulan 71 kDa.

Detector Constant	Average	Peak
$Q_{RI}$/mV	8.921 $\times \;10^{6}$	
$Q_{LALS}$/(mV mol ${\mathrm{cm}}^{-3}$)	32.24	33.19
$Q_{RALS}$/(mV mol ${\mathrm{cm}}^{-3}$)	56.64	57.50

**Table 3:** Results for the molecular weight and intrinsic viscosity obtained from our chromatograms, analyzed with our SECtools programs, compared to nominal data and the MHS parameters found in the literature.

Sample	M, kDa (Nominal)	M, kDa (Found)	[η], mL/g (Found)	[η] mL/g (MHS Equation)
Pul71	70.7	69.5	32.2	33.6
Pul48	48.8	51.3	24.7	21.1
Pul113	113.0	115.7	43.5	45.8
Pul210	210.0	214.4	65.3	69.3
Pul805 (a)	805	860 (a)	170 (a)	170.0
Pul48 (a)	48.8	46.1 (a)	25.1 (a)	21.1

(a) Values estimated in the chromatograms of the bimodal sample Pul48+805.

## Data Availability

Data are contained within the article.
